# Serum Potassium and Mineralocorticoid Receptor Antagonists As Modifiers of Atrial Arrhythmia Recurrence After Catheter Ablation for Atrial Fibrillation: A Narrative Review With Systematic Search and Mechanistic Synthesis

**DOI:** 10.7759/cureus.114995

**Published:** 2026-08-22

**Authors:** Shunichiro Tomita, Takuya Kishi

**Affiliations:** 1 Department of Graduate School of Medicine (Cardiology), International University of Health and Welfare, Okawa, JPN

**Keywords:** atrial fibrillation, catheter ablation, mineralocorticoid receptor antagonist, recurrence, serum potassium

## Abstract

Atrial arrhythmia recurrence after catheter ablation for atrial fibrillation (AF) remains common despite advances in pulmonary vein isolation, mapping, lesion delivery, and rhythm monitoring. Contemporary guidelines emphasize integrated, risk-factor-based care, yet the roles of serum potassium management and mineralocorticoid receptor antagonist (MRA) therapy after ablation remain poorly defined. We aimed to synthesize the clinical and mechanistic evidence on whether serum potassium, potassium-management strategies, and MRA therapy (steroidal: spironolactone, eplerenone; nonsteroidal: esaxerenone, finerenone) modify recurrence, and to derive a testable mechanistic framework. Considering these backgrounds, we conducted a narrative review supported by a systematic literature search, prepared with reference to the PRISMA 2020 principles for transparency of search and selection and, because clinical heterogeneity precluded meta-analysis, the Synthesis Without Meta-analysis (SWiM) guideline. PubMed/MEDLINE and public professional society repositories were searched from inception to 6 June 2026, supplemented by hand searching; eligibility was prespecified using a PICOS framework. Study selection and extraction were performed in duplicate. The risk of bias was assessed using RoB 2 (randomized trials) and ROBINS-I (non-randomized studies). Findings were synthesized narratively across a prespecified evidence hierarchy (direct, indirect, mechanistic). Direct evidence was limited to five studies and was constrained by heterogeneity, small sample sizes in the MRA studies, and moderate-to-serious risk of bias in most non-randomized comparisons. One registry cohort (n = 4,838) reported a U-shaped association between preprocedural potassium and one-year recurrence, with the lowest observed risk at 4.41-4.60 mmol/L; however, this observational association does not establish an optimal therapeutic target or benefit from supplementation. Among four small/heterogeneous MRA studies, eplerenone and a single-center spironolactone randomized trial showed favorable recurrence signals, whereas retrospective esaxerenone showed no recurrence reduction despite improvements in secondary biomarker and remodeling measures; another study used renal sympathetic denervation as an active comparator and therefore did not establish MRA benefit over usual care. These findings should be interpreted cautiously because of study limitations, imprecision, confounding by indication, and variable recurrence ascertainment. Indirect evidence from non-ablation populations and mechanistic studies supports biological plausibility for interactions among potassium homeostasis, mineralocorticoid signaling, renal function, and atrial remodeling, but cannot validate a post-ablation treatment strategy. Accordingly, the proposed ionic-renal-aldosterone-fibrosis axis should be regarded as a hypothesis-generating framework rather than a clinically validated mechanism. Current evidence supports avoiding hypokalemia and hyperkalemia, correcting reversible disturbances, and prescribing MRAs for established indications with appropriate monitoring; it does not justify routine potassium supplementation or MRA use solely to prevent post-ablation recurrence. Prospective, adequately powered randomized studies with longitudinal potassium assessment and rigorous rhythm monitoring are required before mechanism-based post-ablation interventions can be recommended.

## Introduction and background

Catheter ablation is a rhythm-control procedure that eliminates or electrically isolates tissue responsible for initiating or sustaining atrial fibrillation (AF). Its cornerstone is pulmonary vein isolation (PVI), in which circumferential ablation lesions electrically disconnect the pulmonary veins, a major source of AF triggers, from the left atrium [1-4]. Despite technical advances, recurrent AF, atrial flutter (AFL), or atrial tachycardia remains common after ablation. Long-term single-procedure freedom from atrial arrhythmia is approximately 53% in a systematic review, although outcomes vary substantially according to AF phenotype, procedural strategy, and monitoring intensity [5].

The object of this review is specifically post-ablation atrial arrhythmia recurrence, not AF prevention in the general population. Recurrence may result from pulmonary vein reconnection, gaps in ablation lesions, non-pulmonary-vein triggers, or progression of the atrial substrate [1-5]. Atrial substrate remodeling refers to structural and electrical changes - including atrial enlargement, fibrosis, altered conduction, and changes in refractoriness - that make AF easier to initiate or sustain. Hypertension, heart failure (HF), chronic kidney disease (CKD), obesity, sleep-disordered breathing, inflammation, and autonomic activation can promote this remodeling and may interact with procedural mechanisms [1-6].

Within this post-ablation setting, the review evaluates two related exposures: serum potassium and mineralocorticoid receptor antagonist (MRA) therapy. Potassium directly influences cardiac membrane excitability, repolarization, conduction, and triggered activity [7-9]. Direct post-ablation evidence is limited to one large observational cohort reporting a U-shaped association between preprocedural potassium and one-year recurrence [10]. Evidence linking hypokalemia to incident AF or experimental arrhythmogenesis is therefore considered indirect for the post-ablation question and cannot establish a therapeutic potassium target [7-10].

Mineralocorticoid receptor antagonism refers to pharmacologic blockade of aldosterone signaling at the mineralocorticoid receptor. Steroidal MRAs (spironolactone and eplerenone) and nonsteroidal MRAs (esaxerenone and finerenone) can influence sodium and potassium handling, blood pressure, volume status, inflammation, and fibrotic remodeling, but differ pharmacologically and in their clinical evidence base [11-21]. Four small or near-direct studies have evaluated MRA strategies in relation to AF ablation, with heterogeneous findings [19,22-24]. Meta-analyses of AF prevention and contemporary finerenone trials provide context but do not directly support post-ablation efficacy [11-13,20,21].

The clinically relevant population is adults undergoing catheter ablation for paroxysmal, persistent, or long-standing persistent AF, with particular interest in whether AF subtype and cardiorenal-hypertensive phenotype modify associations with potassium or MRA therapy. HF, CKD, hypertension, diuretic exposure, low or variable potassium, and suspected aldosterone excess may identify biologically distinct subgroups, but none currently defines a validated post-ablation treatment indication [6,14-18,19-26]. The objective is to separate direct post-ablation evidence from indirect evidence of AF prevention and mechanistic evidence, evaluate the certainty and limitations of each tier, and develop testable hypotheses regarding potassium stability and MRA therapy in selected phenotypes.

## Review

Materials and methods

Design and Reporting

This narrative review, supported by a systematic literature search and mechanistic synthesis, follows PRISMA 2020 and the Synthesis Without Meta-analysis (SWiM) reporting guideline [25,26]. We specified the review question, eligibility criteria, search strategy, appraisal approach, evidence hierarchy, and synthesis rules a priori. No pooled meta-analysis was undertaken.

Review Question (Population, Intervention, Comparator, Outcome, and Study Design (PICOS))

The population was adults undergoing catheter ablation for paroxysmal, persistent, or long-standing persistent AF; exposures/interventions were serum potassium concentration, potassium-management strategies, and MRA therapy; comparators were alternative potassium categories, usual care, no MRA, or alternative upstream therapy. The primary outcome was recurrent AF, AFL, or atrial tachycardia after ablation, preferentially after the study-defined blanking period. Secondary outcomes included early recurrence, AF burden, repeat ablation, cardioversion, remodeling measures, biomarkers, renal outcomes, hypokalemia/hyperkalemia, and treatment discontinuation.

Information Sources and Search

We searched PubMed/MEDLINE and publicly available professional-society guideline repositories from inception to 6 June 2026, supplemented by hand searches of reference lists. Search terms covered AF, catheter ablation, recurrence, potassium, hypokalemia/hyperkalemia, potassium supplementation, MRAs, aldosterone/renin-angiotensin-aldosterone system (RAAS), atrial fibrosis, renal function, and CKD. Restriction to English-language, PubMed-indexed clinical literature was pragmatic and may introduce language and database-selection bias [25,26].

Eligibility and Study Selection

Eligible clinical sources were English-language, PubMed-indexed RCTs and prospective or retrospective observational studies addressing the PICOS question. Systematic reviews, meta-analyses, mechanistic/translational studies, and public professional-society documents were eligible as indirect or contextual evidence. Both authors screened and made eligibility decisions independently, and resolved disagreements through discussion and consensus.

Evidence Hierarchy and Mechanistic Evidence

We prespecified three tiers: direct post-ablation clinical evidence; indirect clinical evidence from AF or relevant cardiovascular populations not defined exclusively by ablation; and mechanistic evidence. We selected mechanistic studies for direct relevance to a biological step in the proposed pathway and appraised them narratively for experimental context, consistency, directness, and applicability to the human post-ablation atrium. RoB 2 and ROBINS-I were not applied to experimental mechanistic studies. We used mechanistic evidence for biological interpretation and hypothesis generation, not to establish clinical efficacy.

Data Items, Recurrence Harmonization, and Quantitative Derivations

For direct studies, we extracted design, sample size, AF phenotype, ablation strategy, exposure/intervention and comparator, follow-up, blanking period, recurrence definition, rhythm-monitoring strategy, efficacy outcomes, and safety outcomes. We harmonized recurrence conceptually rather than by imposing an unmeasured common threshold: we retained each study's prespecified definition and considered monitoring modality and intensity when interpreting results. We treated early recurrence, post-blanking recurrence, and AF burden as distinct outcome constructs.

Where a primary report provided group sample sizes and AF-free proportions but not recurrence counts or comparative effect measures, recurrence events were approximated as n multiplied by (1 - AF-free proportion) and rounded to the nearest whole event. Descriptive risk ratios (RRs), odds ratios (ORs), and risk differences (RDs) were reconstructed from the resulting 2 × 2 table; 95% CIs were calculated using conventional large-sample methods on the log scale for RR/OR and the normal approximation for RD. We labeled these reconstructed estimates as approximate, used them only to aid interpretation, and did not treat them as substitutes for published adjusted estimates. We did not perform a pooled estimate, meta-regression, or hypothesis test combining studies. We interpreted the active-comparator study separately so conclusions did not depend on an inappropriate MRA vs. control assumption.

Risk-of-Bias and Certainty Assessment

Table 1 summarizes the PICOS framework. We appraised RCTs with RoB 2 and non-randomized clinical studies with ROBINS-I [27,28]. Appraisal considered baseline imbalance, treatment-selection bias, cointerventions, adjustment for important confounders, missing outcome data, selective reporting, and differences in rhythm ascertainment. Both authors assessed risk independently; after discussion, they handled unresolved disagreement conservatively by retaining the higher-risk judgment. We also qualitatively assessed outcome-level certainty using Grading of Recommendations Assessment, Development and Evaluation (GRADE) principles across risk of bias, inconsistency, indirectness, imprecision, and potential publication/reporting bias.

**Table 1:** PICOS framework and evidence hierarchy AF, atrial fibrillation; AFL, atrial flutter; LA, left atrium; MRA, mineralocorticoid receptor antagonist; PICOS, Population, Intervention, Comparator, Outcome, and Study Design

Element	Definition used in this review
Population	Adults undergoing catheter ablation for paroxysmal, persistent, or long-standing persistent AF.
Exposure/intervention	Serum potassium level; potassium-management strategy; MRA therapy - steroidal (spironolactone, eplerenone) or nonsteroidal (esaxerenone, finerenone).
Comparator	Alternative potassium category, usual care, no MRA therapy, or alternative upstream therapy.
Primary outcome	Recurrent AF, AFL, or atrial tachycardia after ablation, preferably after the blanking period.
Secondary outcomes	Early recurrence, AF burden, repeat ablation, cardioversion, LA remodeling, biomarkers, renal outcomes, hypokalemia, hyperkalemia, discontinuation.
Evidence hierarchy	Direct ablation evidence prioritized; indirect AF evidence and mechanistic evidence used to support biological interpretation.

Results

Study Selection and Evidence Map

**Figure 1:**
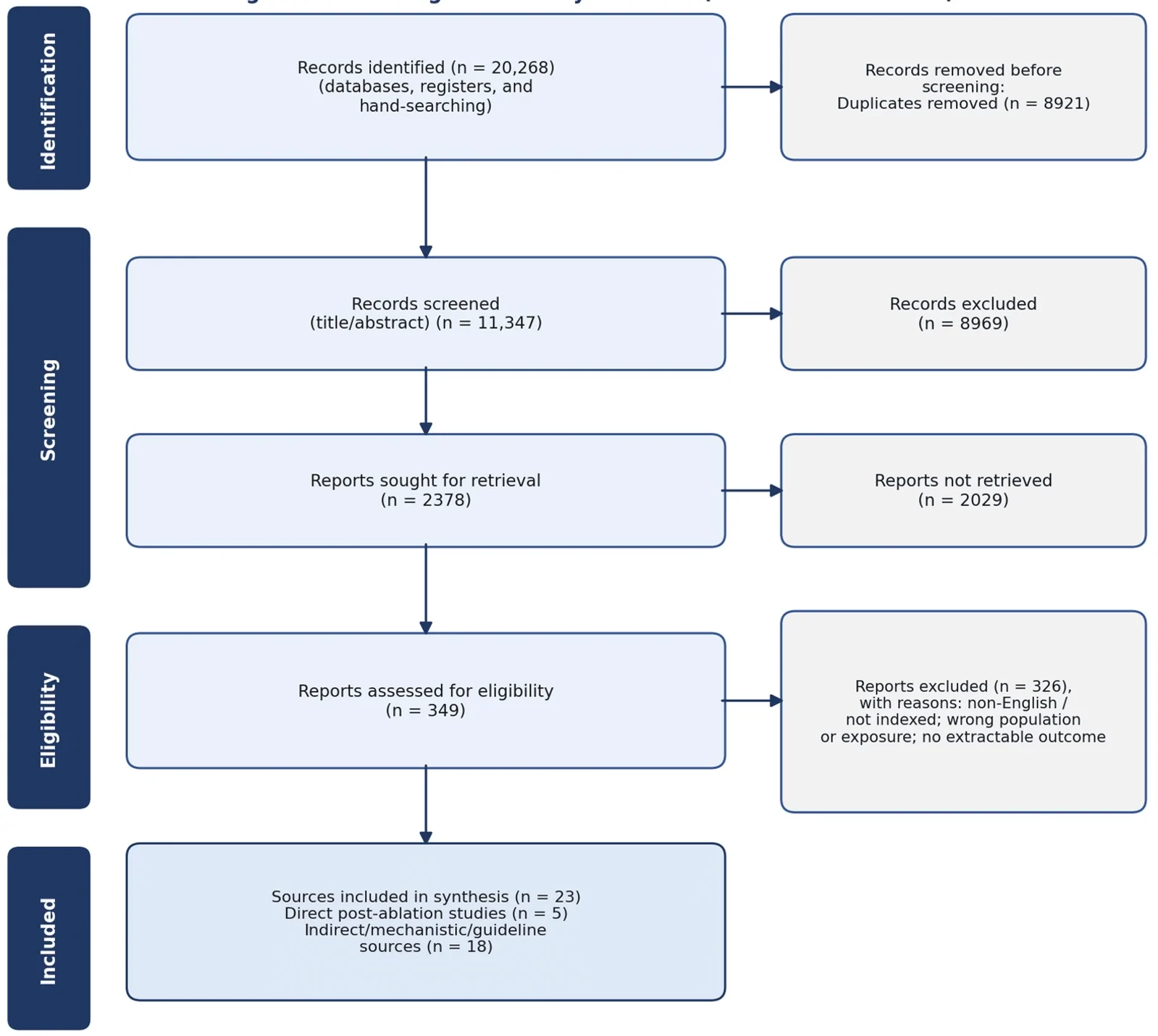
Flow diagram of study identification, screening, eligibility, and inclusion, presented in PRISMA 2020 format Of 20,268 records identified and 11,347 screened after removal of 8,921 duplicates, 2,378 reports were sought, 349 were assessed for eligibility, and 23 sources were included (five direct post-ablation studies; 18 indirect, mechanistic, or guideline sources).

The search yielded 20,268 records; after removing 8,921 duplicates, we screened 11,347, excluded 8,969, and sought retrieval of 2,378 reports. Of these, 2029 were not retrieved, 349 were assessed for eligibility, and 326 were excluded with reasons, leaving 23 sources for synthesis: five direct post-ablation studies and 18 indirect, mechanistic, or guideline sources (Figure 1). Four further references provide methodological or reporting guidance and are not counted among the included sources [25-28]. The search identified one large direct study of preprocedural serum potassium and recurrence [10] and four small/near-direct studies of MRA therapy around ablation [19,22-24]; Table 2 summarizes the studies. Indirect and mechanistic sources are integrated. Table 2 presents sample sizes and effect estimates extracted from the primary reports; for Ito et al. and Kiuchi et al., recurrence events and effect estimates were approximated from the reported event rates [12,24]. Importantly, Kiuchi et al. used an active comparator (renal sympathetic denervation), not placebo or usual care, and are therefore handled separately from the MRA vs. control evidence [24].

**Table 2:** Direct and near-direct clinical evidence relevant to post-ablation recurrence Effect estimates use AF recurrence as the event; values < 1 (RR, OR) and < 0 (RD) indicate fewer recurrences in the mineralocorticoid receptor antagonist (MRA) group. All 95% confidence intervals are shown in parentheses. ᵃRecurrence events and effect estimates approximated from reported event rates (Ito et al.; Kiuchi et al.). ᵇKiuchi et al. used an active comparator (RSD), not placebo or usual care; with spironolactone as the index group, RR/OR > 1 indicates more recurrence with spironolactone than with RSD (i.e., RSD was superior). This study is not an MRA vs. control comparison and should be analyzed separately (sensitivity analysis only). Secondary continuous outcomes - Wang et al. 2021: angiotensin II -11.5 pg/mL (p < 0.001), aldosterone 45.7 vs. 60.6 pg/mL (p = 0.016), NT-proBNP 73.5 vs. 110 pg/mL (p = 0.016), left atrial diameter 35.8 vs. 37.2 mm (p = 0.007); Kashiwagi et al. 2025: BNP at six months 50.0 vs. 64.6 pg/mL (p = 0.02), LVMI at one year 95.2 vs. 108.0 g/m² (p < 0.01). AF, atrial fibrillation; CA, catheter ablation; CKD, chronic kidney disease; LVMI, left ventricular mass index; OR, odds ratio; PVI, pulmonary vein isolation; RCT, randomized controlled trial; RD, risk difference; RR, relative risk; RSD, renal sympathetic denervation

Study	Design and population	Groups, n	AF recurrence (rate; p)	Effect estimate for AF recurrence
Yang et al. 2024 [10]	Prospective registry cohort; de novo AF catheter ablation; 1-year recurrence after 3-month blanking.	4,838 (potassium categories)	U-shaped vs.preprocedural potassium; lowest risk at 4.41-4.60 mmol/L.	Restricted cubic spline + Cox; non-linear (no single 2 × 2).
Ito et al. 2013 [22]	Eplerenone vs. no eplerenone after CA for long-standing persistent AF; 24-month follow-up; observational.	Eplerenone 55; control 106 (161)	AF-free 60% vs. 40% (p = 0.011); multivariable Cox p = 0.017.	≈22/55 vs. ≈64/106; RR 0.66 (0.46-0.95); OR 0.44 (0.22-0.85); RD -20.4% (-36.3 to -4.4)ᵃ
Wang et al. 2021 [23]	Single-center RCT; PVI + spironolactone vs. PVI alone.	Spironolactone 102; control 101 (203)	AF-free 85.3% vs.73.3% (p = 0.033).	15/102 vs. 27/101; RR 0.55 (0.31-0.97); OR 0.47 (0.23-0.95); RD -12.0% (-23.1 to -1.0)
Kiuchi et al. 2018 [24]	CKD, resistant hypertension, paroxysmal AF, pacemaker; PVI + spironolactone vs.PVI + RSD (active comparator); 1-year.	Spironolactone 36; RSD 33 (69)	AF-free 36% vs. 61% (p = 0.024); RSD also ↓ AF burden.	≈23/36 vs. ≈13/33; RR 1.62 (0.99-2.65); OR 2.72 (1.03-7.21); RD +24.5% (+1.6 to +47.4)ᵃᵇ
Kashiwagi et al. 2025 [19]	Esaxerenone vs. no esaxerenone after CA for persistent AF + hypertension; retrospective.	Esaxerenone 40; control 117 (157)	1-year AF-free 80% vs.84% (p = 0.60).	8/40 vs. 19/117; RR 1.23 (0.59-2.59); OR 1.29 (0.52-3.23); RD +3.8% (-10.3 to +17.8)

The direct MRA evidence is mixed and, when quantified, modest in size. Steroidal MRAs showed favorable signals-eplerenone (relative risk for recurrence ≈ 0.66, 95% CI 0.46-0.95) and the single-center spironolactone RCT (relative risk 0.55, 95% CI 0.31-0.97) - whereas the nonsteroidal MRA esaxerenone was neutral (relative risk 1.23, 95% CI 0.59-2.59; p = 0.60) [19,22,23]. The fourth study compared spironolactone with renal sympathetic denervation rather than a control, and the denervation arm had fewer recurrences than the spironolactone arm (freedom from AF 61% vs.36%; p = 0.024), so this does not provide evidence of MRA benefit over usual care [24]. Heterogeneity may reflect sample size, MRA pharmacology, phenotype, hypertension severity, renal function, baseline potassium, follow-up duration, ablation strategy, background RAAS inhibition, and monitoring intensity [10,14,19-24]. All direct MRA studies are vulnerable to confounding by indication, because MRAs are prescribed for conditions-HF, hypertension, CKD, and suspected aldosterone excess-that themselves modify recurrence risk [6,14,17,18,27,28].

Indirect clinical evidence supports biological plausibility. The Rotterdam Study associated hypokalemia with incident AF [7]. Experimental studies show that hypokalemia is arrhythmogenic via chamber-specific mechanisms [8]. A major electrophysiologic review explains why both hypokalemia and hyperkalemia destabilize conduction and repolarization [9]. In cardiac surgery, the TIGHT K trial found that a relaxed potassium-supplementation threshold was non-inferior to a high-normal threshold for postoperative AF, cautioning against indiscriminate, aggressive supplementation [29]. Meta-analyses associate MRAs with fewer AF events, including a reduced recurrent AF signal in Alexandre et al. and fewer AF events across randomized trials in Oraii et al., although heterogeneous AF ascertainment limits certainty [11-13]. CKD is a particularly important modifier, increasing recurrence risk and the risk of MRA-related hyperkalemia [6,14,17].

Risk-of-Bias Appraisal

We appraised direct studies using RoB 2 for randomized trials and ROBINS-I for non-randomized studies [29,30]. In addition to confounding by indication and selection bias, the appraisal explicitly considered baseline imbalance, cointerventions, exposure classification, adjustment for clinically important confounders, missing outcome data, selective outcome reporting, and outcome ascertainment. For observational studies, particular attention was paid to whether analyses accounted, where reported, for major determinants of recurrence and treatment selection, including AF phenotype, age, sex, hypertension, heart failure, renal function, left atrial remodeling, baseline potassium, diuretic and background RAAS-inhibitor use, and differences in ablation strategy. Because reporting of these variables and adjustment sets was incomplete or inconsistent across the primary studies, we could not exclude residual confounding, even with multivariable adjustment.

We also considered selective outcome reporting and missing outcome data. The available reports did not provide sufficient information to confidently exclude selective reporting, particularly in small single-center studies, where recurrence, biomarkers, remodeling measures, and safety outcomes were reported variably. Where loss to follow-up, completeness of rhythm surveillance, or prespecified vs. reported outcomes could not be fully reconstructed from the published report, this uncertainty was incorporated into the risk-of-bias judgment rather than assumed to be absent. We therefore did not interpret statistically significant secondary biomarker or remodeling findings as confirmatory evidence of an antiarrhythmic effect when the primary rhythm outcome was neutral or when outcome reporting was incomplete.

Detection bias was a major cross-study concern because rhythm-monitoring intensity and recurrence ascertainment varied across studies. Intermittent scheduled electrocardiography, symptom-triggered recordings, Holter/ambulatory monitoring, and implanted-device interrogation have different sensitivities for asymptomatic or short-duration AF. Consequently, an apparently higher freedom-from-AF rate in a less intensively monitored cohort cannot be assumed to represent a biologically lower AF burden than a rate obtained with continuous or device-based surveillance. We therefore evaluated monitoring modality, frequency, duration, symptom dependence, blanking-period definition, and follow-up completeness as components of outcome-measurement bias. We treated differences in ascertainment as a source of both risk of bias and clinical heterogeneity, and interpreted effect estimates within each study's surveillance strategy rather than comparing them as if recurrence detection were uniform.

The large potassium registry remained at moderate-to-serious risk of bias because its observational design cannot distinguish a causal effect of potassium from confounding by renal function, comorbidity, medication exposure, or other upstream physiology [10]. The non-randomized MRA studies had serious concerns regarding treatment selection, small sample sizes, baseline and phenotype differences, co-interventions, incomplete adjustment, and heterogeneous rhythm surveillance [19,22,24]. The single-center spironolactone RCT provided stronger protection against confounding but raised concerns because the published report could not verify allocation concealment, blinding, missing-data handling, rhythm surveillance, selective reporting, and safety reporting in sufficient detail [23,27]. Table 3 summarizes these judgments, which should be interpreted as review-level assessments based on the information available in the published primary reports.

**Table 3:** Summary risk-of-bias appraisal of direct studies RoB 2, Cochrane risk-of-bias tool for randomized trials; ROBINS-I, Risk Of Bias In Non-randomized Studies of Interventions

Study	Tool/overall	Principal concerns
Yang et al. 2024 [10]	ROBINS-I: moderate-serious	Confounding by indication and residual confounding; potassium as marker of upstream conditions; observational association cannot establish causality of supplementation.
Ito et al. 2013 [22]	ROBINS-I: serious	Non-randomized exposure; small sample; selection by clinical indication; no longitudinal potassium/aldosterone/imaging mechanism data.
Wang et al. 2021 [23]	RoB 2: some concerns	Single-center, modest size; incomplete reporting of allocation/blinding and safety; uncertain generalizability.
Kiuchi et al. 2018 [24]	ROBINS-I: serious	Active comparator (RSD), not placebo/usual care - spironolactone arm had more recurrence; highly selected phenotype; very limited generalizability; high hyperkalemia/renal risk; small sample. Not an MRA vs. control comparison.
Kashiwagi et al. 2025 [19]	ROBINS-I: serious	Retrospective; confounding; endpoint/phenotype dependence; biomarker improvement without rhythm benefit.

To complement study-level risk-of-bias assessment, we added an outcome-level certainty assessment using GRADE principles. Because the evidence was too sparse and heterogeneous for formal meta-analysis, we evaluated certainty qualitatively across risk of bias, inconsistency, indirectness, imprecision, and publication/reporting bias rather than pooling effect estimates. For the association between preprocedural potassium and post-ablation recurrence, we rated certainty as very low: evidence comes from a single observational cohort, with residual confounding and no randomized evidence that modifying potassium changes recurrence [10]. For MRA therapy and post-ablation recurrence, we rated certainty as low overall: one modest single-center RCT suggested benefit, but the remaining studies were small, heterogeneous, predominantly non-randomized, and yielded inconsistent results with important risks of bias and imprecision [19,22-24]. Indirect AF-prevention and mechanistic evidence were not used to upgrade certainty for post-ablation efficacy because they are indirect relative to the review's primary clinical question. Thus, neither evidence stream currently supports a high-certainty causal treatment recommendation.

Serum Potassium and Atrial Arrhythmia Recurrence After Ablation

The direct evidence for potassium is observational and should not be interpreted as defining a therapeutic target. In the registry cohort of 4,838 patients undergoing de novo ablation, preprocedural potassium showed a U-shaped association with one-year recurrence, with the lowest observed risk at 4.41-4.60 mmol/L [10]. This range is therefore an epidemiologic risk nadir within one cohort, not a validated treatment window. The study does not establish that actively moving potassium into this range, or supplementing patients whose values are already within the reference range, reduces recurrence. Any numerical range proposed for intervention requires prospective validation before it can be considered a therapeutic target.

Baseline potassium may also incompletely represent the ionic milieu surrounding ablation. Potassium varies with renal function, acute illness, dietary intake, neurohormonal activation, diuretic exposure, RAAS inhibition, MRA use, and replacement therapy [6-10,14,17,18]. Peri-procedural measurements and longitudinal trajectories may therefore be more informative than a single preprocedural value. Future studies should distinguish baseline concentration from peri-ablation excursions, persistent hypokalemia or hyperkalemia, within-patient variability, and changes associated with medication adjustment.

Concomitant interventions that alter both potassium and arrhythmic susceptibility may also influence observed recurrence. Class III or other QT-prolonging antiarrhythmic drugs may increase the clinical relevance of hypokalemia or hypomagnesemia, whereas sodium-channel blockers interact with conduction reserve [8,9]. Loop and thiazide diuretics, changes in diuretic dose after ablation, initiation or titration of RAAS inhibitors or MRAs, and institutional potassium/magnesium replacement protocols can shift potassium exposure during the blanking period. These factors may confound or modify associations between measured potassium and recurrence and should be prospectively recorded rather than treated as background therapy.

Mechanistic interpretation also requires caution. Some principles of potassium-dependent membrane excitability and conduction are shared across cardiac tissue, but atrial and ventricular electrophysiology are not interchangeable. Chamber-specific ion-channel expression, action-potential morphology, calcium handling, structural substrate, and responses to hypokalemia can produce different arrhythmogenic mechanisms [8,9]. Experimental evidence specifically demonstrates distinct effects of hypokalemia in atrial and ventricular myocytes [8]. Accordingly, ventricular electrophysiologic observations can inform general biological plausibility but should not be assumed to quantitatively predict post-ablation atrial behavior.

To move beyond a single potassium value, we propose “potassium time-in-zone” as a research construct rather than a clinically established metric. In prospective cohorts, potassium could be measured at standardized time points before ablation, immediately peri-procedurally, during the blanking period, after relevant medication changes, and during longer-term follow-up. Time-in-zone could then be estimated from serial measurements as the proportion of observed follow-up time within a prespecified candidate interval, using interpolation between measurements where appropriate. Define candidate zones a priori and test them in sensitivity analyses rather than deriving and validating them in the same dataset. Complementary metrics should include time below 3.5 and 4.0 mmol/L, time above 5.0 and 5.5 mmol/L, absolute variability, and clinically significant excursions. Validation would require demonstration of reproducibility, incremental prognostic value beyond baseline potassium and established recurrence predictors, temporal association with continuously or intensively monitored AF burden, and ultimately prospective evidence that a protocol designed to improve potassium stability improves clinical outcomes without increasing hyperkalemia. Until such validation is available, potassium time-in-zone remains hypothesis-generating.

Mineralocorticoid Receptor Antagonists After AF Ablation

Steroidal vs. nonsteroidal MRAs: Current evidence does not establish a class effect of MRAs on post-ablation recurrence. Spironolactone and eplerenone are steroidal MRAs, whereas esaxerenone and finerenone are nonsteroidal agents with differences in receptor selectivity, tissue distribution, pharmacokinetics, blood-pressure effects, renal handling, and potassium pharmacodynamics [14,19-21]. The direct ablation literature is too small and heterogeneous to determine whether divergent findings represent true agent-specific effects, differences in patient phenotype and study design, or chance. Accordingly, favorable findings with one MRA should not be extrapolated to another, and neither steroidal nor nonsteroidal MRAs can currently be regarded as having a proven class-wide anti-recurrence effect after AF ablation.

Hyperkalemia risk should likewise not be assumed to be identical across agents or populations. Risk depends on the individual MRA, dose and titration, baseline potassium, eGFR, diabetes and HF status, concomitant angiotensin-converting enzyme inhibitor (ACEI)/angiotensin receptor blocker (ARB)/angiotensin receptor-neprilysin inhibitor (ARNI) therapy, diuretics, potassium supplements or salt substitutes, and monitoring intensity [14,17,18]. The available post-ablation studies are not sufficiently powered or consistently reported to directly compare hyperkalemia rates among spironolactone, eplerenone, esaxerenone, and finerenone. Therefore, agent selection in current practice should follow established indications and drug-specific renal/potassium safety guidance rather than presumed antiarrhythmic equivalence.

Mechanistic rationale and direct ablation studies: The four MRA-related ablation studies are clinically heterogeneous, which substantially limits direct comparison [19,22-24]. They enrolled different AF phenotypes and comorbidity profiles, ranging from long-standing persistent AF to persistent AF with hypertension and a highly selected population with CKD, resistant hypertension, paroxysmal AF, and pacemakers. These differences imply markedly different burdens of pulmonary-vein triggers, atrial remodeling, neurohormonal activation, renal risk, and potential responsiveness to MR blockade.

Procedural and follow-up differences further complicate synthesis. Ablation techniques, adjunctive lesion strategies, background therapies, follow-up duration, blanking-period handling, and rhythm-monitoring intensity were not uniform [19,22-24]. A study using more intensive or device-based surveillance may detect substantially more asymptomatic recurrence than one relying on intermittent or symptom-driven assessment. Thus, inconsistent findings may reflect differences in phenotype, procedural era and technique, surveillance, or follow-up rather than pharmacologic differences alone.

Biomarker and structural changes should not be regarded as validated surrogate endpoints for recurrent atrial arrhythmia. Reductions in BNP/NT-proBNP, blood pressure, left ventricular mass index, or left atrial diameter may indicate hemodynamic or remodeling effects, but current evidence does not show that treatment-induced changes in these measures reliably predict reduced post-ablation AF burden or recurrence. The neutral rhythm result despite improved BNP and LVMI with esaxerenone is an important example [19]. Such secondary outcomes are therefore mechanistically informative but cannot substitute for rigorously ascertained rhythm endpoints.

Statistical power is another major limitation. Except for the large observational potassium cohort, the direct MRA studies were small, and confidence intervals around recurrence effects were correspondingly wide [19,22-24]. Positive findings may overestimate effects in small studies, whereas neutral findings cannot reliably exclude clinically meaningful benefit or harm. Interpret the evidence by effect estimates and precision rather than statistical significance alone.

Observational comparisons are also vulnerable to treatment-selection bias beyond conventional confounding by indication. Clinicians may preferentially prescribe an MRA according to blood pressure, renal function, potassium, HF status, edema, concomitant therapy, perceived adherence, or severity of atrial remodeling; these factors can also affect follow-up intensity and recurrence risk. Baseline imbalance, differential cointerventions, medication changes during follow-up, and incompletely measured variables may persist despite multivariable adjustment. Consequently, residual confounding remains plausible and causal interpretation of observational MRA associations is not justified [19,22,24,27,28]. 

Against this background, Ito et al. reported greater AF-free survival with eplerenone after ablation for long-standing persistent AF, but the observational design and incomplete mechanistic measurements preclude causal attribution [22]. Wang et al. reported a favorable recurrence signal with spironolactone in a modest single-center RCT, but external replication with contemporary ablation and rigorous rhythm surveillance is required [23]. Kiuchi et al. used renal sympathetic denervation as an active comparator in a highly selected phenotype and therefore do not estimate MRA efficacy vs. usual care [24]. Kashiwagi et al. found no recurrence reduction with esaxerenone despite improvements in BNP and LVMI, reinforcing that biomarker improvement does not establish rhythm benefit [19]. Collectively, these studies provide preliminary, heterogeneous signals rather than a clinically validated post-ablation MRA strategy.

Indirect trial-level and contemporary nonsteroidal-MRA evidence: Indirect evidence supports biological plausibility but has important limitations in external validity for patients after AF ablation [11-13,20,21]. The contributing populations span different baseline cardiovascular risks, including HF, CKD, diabetes, hypertension, and broader cardiovascular cohorts, and differ substantially in baseline AF status, structural heart disease, renal function, background RAAS therapy, and intensity of rhythm ascertainment. Absolute and relative effects observed in these populations may therefore not apply to an ablated population in whom pulmonary-vein reconnection, lesion-related substrate, and post-procedural healing contribute to recurrence.

The pooled estimates from prior MRA meta-analyses should also not be interpreted as demonstrating a uniform class effect across clinical settings. The included trials differed in the MRA used, treatment indication, AF phenotype, whether AF was incident or recurrent, and whether AF was a primary or secondary outcome [11-13]. Consequently, aggregate estimates may be disproportionately influenced by particular clinical populations or intervention types, obscuring effect modification. The available review-level evidence does not permit a reliable conclusion that the pooled benefit is equally attributable to each MRA or equally applicable to post-ablation recurrence.

Contemporary finerenone analyses add higher-quality randomized evidence for atrial arrhythmia outcomes in cardio-kidney-metabolic populations but remain indirect for the present question [20,21]. In FINE-HEART, finerenone reduced new-onset AF/AFL among participants free of AF/AFL at baseline [20], whereas the FINEARTS-HF analysis addressed HF populations and did not establish prevention of recurrent AF after ablation [21]. These data identify finerenone as a biologically and clinically relevant candidate for future trials, but differences in baseline risk, outcome definition, and procedural context preclude direct extrapolation to post-ablation efficacy.

Aldosterone, primary aldosteronism (PA), and the limits of MR blockade: PA provides clinically important evidence that aldosterone excess is associated with the risk of atrial arrhythmia, but it does not yet define a validated biomarker strategy for selecting post-ablation patients for MRA therapy [15,16]. Candidate markers include suppressed renin, aldosterone concentration, the aldosterone-renin ratio, spontaneous or diuretic-associated hypokalemia, resistant hypertension, adrenal abnormalities, and evidence of disproportionate cardiac remodeling. However, thresholds developed for PA diagnosis or cardiovascular risk should not automatically be assumed to identify an MRA-responsive post-ablation phenotype. HF, CKD, obesity, sodium intake, concomitant RAAS therapy, and diuretics can also alter renin, aldosterone, potassium, and treatment response.

Future studies should therefore prospectively define both the aldosterone phenotype and adequacy of MR blockade. Potential pharmacodynamic measures include changes in renin from baseline, correction of hypokalemia, blood pressure and volume responses, changes in aldosterone-renin indices, and prespecified biochemical or clinical evidence of adequate receptor blockade, while simultaneously monitoring potassium and renal safety [14-18]. In PA, normalization or unsuppression of renin during medical therapy has been proposed as a marker of more adequate MR blockade, but whether this approach predicts lower post-ablation recurrence remains to be validated prospectively [16]. Drug adherence, dose intensity, concomitant RAAS therapy, sodium intake, and renal function should also be recorded because apparent treatment failure may reflect inadequate exposure rather than failure of the biological target.

The clinical message is therefore straightforward: MRA efficacy likely depends on selecting patients in whom mineralocorticoid signaling is relevant and achieving adequate MR blockade safely. Current evidence does not support the assumption that all MRAs, at any dose, provide equivalent protection against aldosterone-mediated atrial remodeling. This principle supports phenotype-enriched prospective trials rather than routine class-wide MRA use after AF ablation.

RAAS context, mechanistic ambiguity, and safety: Post-ablation recurrence should not be viewed as the product of independent competing mechanisms. Pulmonary vein reconnection can restore a trigger source, whereas atrial fibrosis, inflammation, autonomic remodeling, and ionic instability determine whether those triggers propagate and sustain recurrent AF [3,4,7-9,11-18]. These processes may therefore interact: reconnection may be clinically silent in a relatively healthy atrium but become arrhythmogenic in a fibrotic, electrically heterogeneous substrate, while extensive atrial cardiomyopathy may sustain AF even when PVI remains durable. Conversely, neurohormonal or electrolyte-directed therapy cannot be expected to compensate for a critical lesion gap or an active non-pulmonary-vein trigger. This interaction helps explain why the same biochemical intervention can have different effects depending on procedural durability and underlying atrial substrate.

The background cardiorenal treatment regimen may further modify both efficacy and safety. Concomitant ACEI/ARB/ARNI therapy can alter aldosterone signaling, blood pressure, renal hemodynamics, and potassium balance; when combined with an MRA, it may strengthen neurohormonal blockade but also increase the need for potassium and renal-function surveillance [14,17,18]. Sodium-glucose cotransporter-2 (SGLT2) inhibitors are increasingly used in patients with HF, CKD, and diabetes and may alter volume status, renal function, and the clinical context in which an MRA is prescribed [17]. Their concomitant use could therefore influence both apparent MRA efficacy and tolerability. However, whether SGLT2 inhibition modifies the effect of an MRA on post-ablation AF recurrence remains unclear. Future studies should prespecify ACEI/ARB/ARNI and SGLT2-inhibitor use, record changes during follow-up, and evaluate treatment interactions together with serial eGFR and potassium rather than treating these drugs as unmeasured background therapy.

Timing also matters. Early recurrence during the blanking period may reflect inflammation, edema, autonomic fluctuation, lesion maturation, electrolyte shifts, and medication changes, whereas later recurrence may increasingly reflect pulmonary vein reconnection, progressive atrial cardiomyopathy, non-pulmonary-vein triggers, or durable channels through scar [3-5]. Potassium instability and MR activation could modify susceptibility in either phase, but their contribution likely depends on the simultaneous presence of procedural and structural drivers. Trials should therefore distinguish early from late recurrence and incorporate information on procedural durability, atrial substrate, and longitudinal biochemical exposure.

Renal function modifies and mediates this therapeutic balance [6,14,17]. CKD is associated with inflammation, oxidative stress, volume overload, RAAS activation, electrolyte instability, and vascular stiffness, and it also increases susceptibility to hyperkalemia during MR blockade [6,14,17]. Accordingly, patients with CKD may simultaneously have a stronger biological rationale for upstream therapy and a narrower safety margin. Renal-safety algorithms, serial potassium assessment, and careful documentation of concomitant RAAS-modifying and diuretic therapies are therefore essential in future post-ablation studies.

Finally, first recurrence is an incomplete rhythm endpoint. A patient with a single brief AF episode detected during intensive monitoring is biologically and clinically different from a patient with frequent or prolonged recurrent episodes. An MRA may not completely prevent AF recurrence, but it could still provide clinically meaningful benefit if it reduces the frequency or duration of recurrent episodes and thereby lowers overall AF burden. Future trials should therefore evaluate both time to first recurrence and AF burden, including episode frequency and duration, preferably with high-density or continuous rhythm monitoring and temporal linkage to potassium excursions and medication changes [3,4,7-10].

Integrated Mechanistic Synthesis: The Ionic-Renal-Aldosterone-Fibrosis Axis

**Figure 2:**
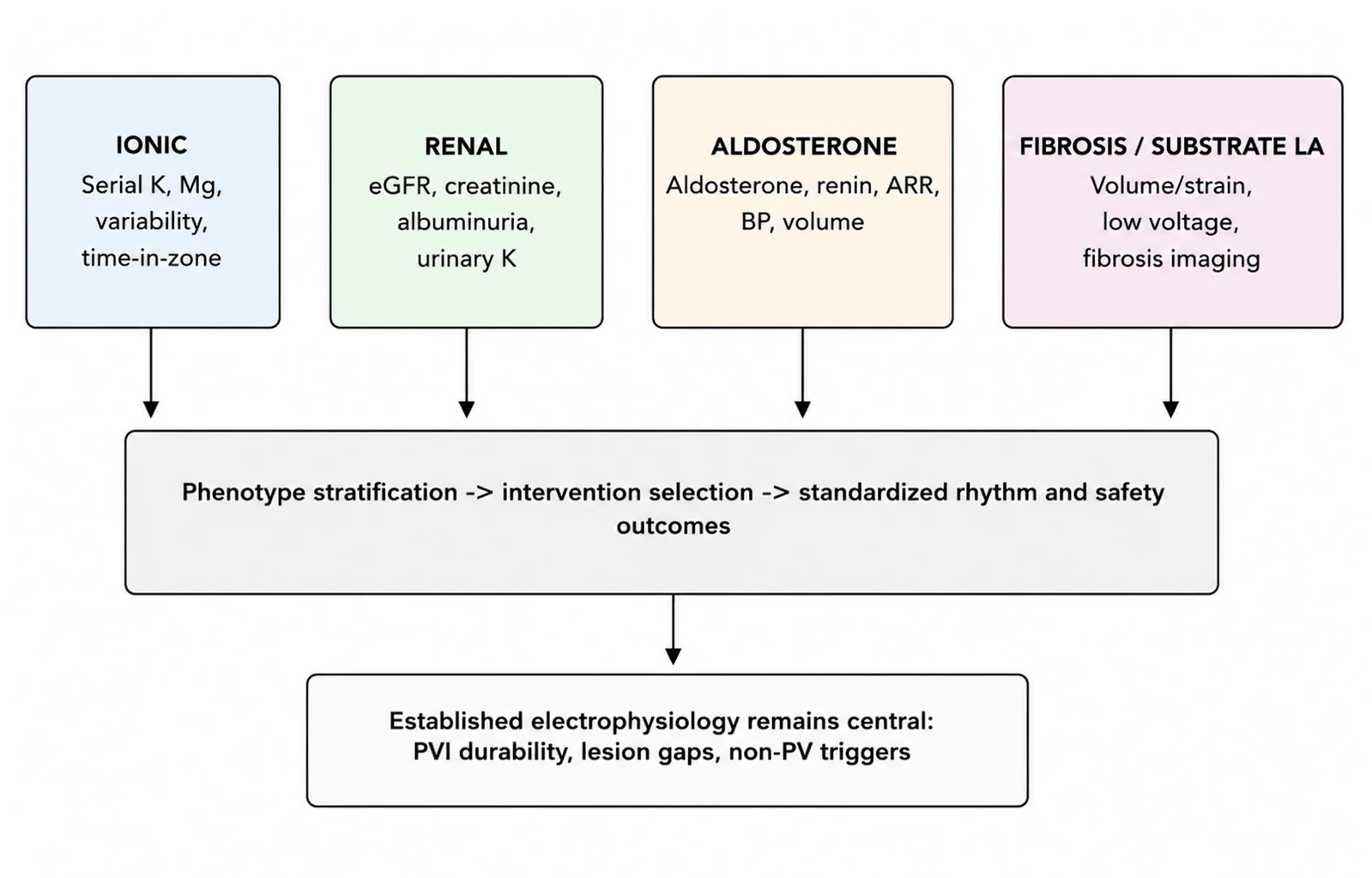
Hypothesis-generating ionic-renal-aldosterone-fibrosis framework linking measurable domains to phenotype stratification, intervention selection, and rhythm/safety outcomes ARR: aldosterone-to-renin ratio; BP: blood pressure; eGFR: estimated glomerular filtration rate; LA: left atrium; PV: pulmonary vein

The evidence can be summarized as a hypothesis-generating four-domain framework rather than as a proven causal pathway (Figure 2). The ionic domain can be measured with serial potassium, magnesium, acid-base status, potassium variability, and candidate potassium time-in-zone metrics. The renal domain can be characterized by creatinine/eGFR, albuminuria, urinary potassium where available, and longitudinal renal-function change. The aldosterone domain can be characterized by plasma aldosterone, renin, aldosterone-renin ratio, blood pressure, volume status, and pharmacodynamic renin response during MR blockade. The fibrosis/substrate domain can be assessed with LA volume and strain, electroanatomic low-voltage extent and conduction properties, atrial late gadolinium enhancement where feasible, and collagen-turnover biomarkers. None is currently validated as a predictor of post-ablation response to potassium intervention or MRA therapy.

This framework is intended to guide testable future studies rather than repeat the individual mechanisms discussed above. Widely available variables could stratify patients according to potassium instability, renal reserve, HF/hypertension phenotype, and atrial remodeling; specialized aldosterone, imaging, or mapping biomarkers could be embedded in mechanistic substudies. Intervention selection would then be linked to the abnormal domain being tested, while established electrophysiologic causes of recurrence - including pulmonary vein reconnection, lesion gaps, and non-pulmonary-vein triggers - remain evaluated conventionally. Outcomes should connect biological change to standardized rhythm endpoints, especially AF burden, while simultaneously measuring renal and electrolyte safety. Because MRAs are preferentially prescribed to patients with HF, CKD, resistant hypertension, and suspected aldosterone excess, residual confounding is difficult to eliminate in observational studies even after multivariable adjustment; this supports phenotype-enriched randomized testing.

Clinical Implications for Current Practice

Current evidence does not support routine potassium supplementation or routine MRA prescription solely to prevent post-ablation recurrence [7-24,29]. The most reasonable candidates for intensified potassium and renal-neurohormonal assessment are patients in whom electrolyte instability or cardiorenal-neurohormonal disease is already clinically plausible: those with prior or current hypo- or hyperkalemia, CKD, HF, resistant or difficult-to-control hypertension, diuretic use, concomitant RAAS-modifying therapy, suspected PA, low or variable potassium, recurrent volume disturbance, or medication changes likely to alter potassium or renal function [6-9,14-18]. Persistent or long-standing AF and marked LA remodeling may warrant greater attention to substrate and comorbidity, but they do not, by themselves, indicate potassium targeting or MRA therapy. In lower-risk patients with stable renal function and electrolytes, routine guideline-supported laboratory surveillance is generally more appropriate than intensified testing.

The proposed approach should remain feasible and risk-based rather than becoming a universal high-frequency laboratory program. Many patients already have potassium and creatinine/eGFR obtained before ablation and, when clinically indicated, after changes in MRAs, RAAS inhibitors, or diuretics [1,2,6,14,17,18]. Additional testing could therefore focus on the early post-ablation period, clinically important medication changes, acute illness, HF decompensation, or previously documented electrolyte instability. This strategy may limit unnecessary blood draws, cost, patient burden, and clinician workload. More intensive serial testing, aldosterone-renin phenotyping, or potassium time-in-zone assessment should currently be regarded as research-oriented approaches whose feasibility, cost-effectiveness, and incremental clinical value require prospective evaluation.

Importantly, the present review does not recommend post-ablation treatment beyond established guideline-supported indications. Correction of clinically relevant hypokalemia or hyperkalemia, evaluation of reversible causes, renal function surveillance, and use of MRAs for established indications remain standard components of clinical management [1,2,6,14,17,18]. Routine potassium supplementation to a fixed high-normal target, routine aldosterone-renin testing in all ablation patients, and initiation of an MRA solely to prevent AF recurrence remain investigational considerations rather than current recommendations.

Intensified monitoring and intervention can themselves cause harm. Repeated laboratory testing may increase cost, inconvenience, false-positive findings, and downstream testing. Reactive medication changes based on isolated potassium values may destabilize blood pressure, volume status, or renal function. Potassium replacement can cause hyperkalemia, particularly in CKD or during RAAS/MRA therapy, whereas excessive reduction of diuretics may worsen congestion. Conversely, overcorrection of hyperkalemia or unnecessary withdrawal of prognostically beneficial cardiorenal therapy may also be harmful. Laboratory results should therefore be interpreted as trends within the clinical context, and medication or electrolyte adjustments should follow established renal and electrolyte safety guidance rather than an unvalidated rhythm-prevention target.

Potassium optimization alone is unlikely to prevent AF recurrence, and MRAs should not be prescribed routinely solely for recurrence prevention. Nevertheless, assessment of potassium homeostasis, renal function, and clinically relevant mineralocorticoid activity can form part of comprehensive AF management when indicated by the patient's phenotype. At present, these considerations should complement, not replace or extend beyond, established electrophysiologic and guideline-supported cardiorenal care [1,2,6-24,29,30].

Reframing the Research Question: From Lesion-Centered to Substrate- and Milieu-Centered Recurrence

Conventional evaluation of recurrent AF after ablation appropriately begins with established electrophysiologic mechanisms: pulmonary vein reconnection, gaps in ablation lines, non-pulmonary-vein triggers, and progressive atrial substrate [3,4]. The atrium is closely interconnected with the renal, endocrine, metabolic, inflammatory, and autonomic systems. The proposed framework does not replace this approach. This conceptual framework also has important implications for future clinical trial design. Rather, it asks whether systemic factors help determine whether a residual or recovered trigger actually produces sustained recurrent AF. For example, pulmonary vein reconnection may provide the trigger, while uncontrolled hypertension, atrial fibrosis, volume overload, or electrolyte instability may make the atrium more capable of sustaining the arrhythmia. Conversely, correcting potassium alone would not be expected to repair a pulmonary vein reconnection or an incomplete lesion.

This raises a more direct research question: after characterizing the procedural mechanism, can correcting a measurable, modifiable systemic abnormality reduce subsequent AF burden in appropriately selected patients? Rather than enrolling all post-ablation patients, future trials should focus on biologically relevant subgroups. One example would be patients with low or fluctuating potassium levels, preserved renal safety, hypertension or aldosterone activation, and atrial remodeling.

Examples include recurrent or variable low potassium in a patient receiving diuretics; suppressed renin or resistant hypertension suggesting mineralocorticoid receptor activation; CKD requiring particularly careful potassium safety assessment; or marked LA remodeling suggesting a substrate in which systemic remodeling may be relevant. Such treatment would remain complementary to repeat electrophysiologic evaluation or re-ablation when reconnection or another procedural target is present.

Phenotype-guided trials nevertheless introduce practical challenges. Renin and aldosterone measurements are affected by medications, sodium intake, posture, renal function, and assay methods; advanced atrial fibrosis imaging and electroanatomic substrate measurements are not uniformly available; and strict enrichment criteria may slow recruitment and reduce generalizability. A pragmatic strategy would therefore use widely available variables - serial potassium, eGFR, blood pressure, HF status, diuretic exposure, and echocardiographic LA remodeling - for core stratification, with aldosterone-renin testing, cardiac magnetic resonance, or detailed mapping incorporated in mechanistic substudies where feasible. Standardize eligibility criteria and biomarker collection prospectively, and test feasibility before large efficacy trials.

A practical recurrence assessment can therefore proceed in parallel rather than as a choice between “lesion” and “milieu”: characterize the recurrent rhythm and AF burden; assess whether pulmonary vein reconnection or another electrophysiologic target is likely; review blood pressure, HF and volume status, renal function, potassium and magnesium trajectories, and medications; and investigate mineralocorticoid excess when clinically indicated. This structured approach is intended as a framework for research and comprehensive evaluation, not as a validated post-ablation treatment algorithm. Before considering repeat ablation, clinicians should systematically evaluate potentially reversible factors, including electrolyte abnormalities, hypertension, obesity, sleep apnea, renal dysfunction, and aldosterone excess. This structured assessment should complement, not replace, conventional electrophysiological evaluation.

Proposed Research Agenda

A definitive research program should progress from prospective phenotyping to randomized testing [18-24,29,30]. Initial cohorts should obtain serial potassium before ablation, during the blanking period, after clinically important medication changes, and during longer-term follow-up, together with magnesium, creatinine/eGFR, albuminuria, diuretic and ACEI/ARB/ARNI/MRA exposure, supplements and salt substitutes, HF status, blood pressure, antiarrhythmic therapy, and rhythm-monitoring intensity [6-18]. These data would determine whether potassium trajectories, variability, or time-in-zone add prognostic information beyond a single baseline measurement and would provide empirical distributions needed to design an intervention.

The rationale for a future potassium-zone intervention would be to reduce clinically important potassium excursions and variability rather than to force potassium toward the observational 4.41-4.60 mmol/L risk nadir [10]. A protocol could use prespecified safety boundaries and standardized responses to low or high values through review of causative medications, magnesium deficiency, dietary factors, diuretic dosing, and clinically indicated replacement. Adherence should be measured at both patient and protocol levels using scheduled laboratory completion, medication reconciliation, pill counts or electronic adherence measures where feasible, documentation of prescribed replacement, and the proportion of protocol-directed actions actually completed. Serial potassium values would provide an objective measure of whether the intervention changed potassium exposure.

A phenotype-enriched randomized trial could use a 2 × 2 factorial design comparing usual potassium care vs.protocolized potassium-stability management and MRA therapy vs. no study MRA [6-24]. Sample size should be based on a prospectively specified, clinically meaningful treatment effect and a contemporary post-blanking event rate measured with the intended monitoring strategy, rather than on the small effects observed in existing studies. For example, if the control recurrence rate were approximately 25-30% at one year, detecting a modest relative reduction would generally require several hundred participants per main comparison, with inflation due to loss to follow-up, factorial interaction testing, and lower-than-expected event rates. Perform a formal calculation only after pilot data establish the expected event rate and achievable effect size under standardized monitoring.

Treatment contamination and crossover require explicit control. Randomization should be stratified by AF type, eGFR, baseline potassium, HF phenotype, diuretic use, and, where available, aldosterone-renin phenotype. Study MRA initiation, discontinuation, dose changes, non-study potassium supplementation, and major diuretic or RAAS-therapy changes should be prospectively recorded. Protocols should define when clinically necessary rescue treatment overrides randomized assignment. The primary analysis should remain intention-to-treat, with prespecified per-protocol and as-treated sensitivity analyses and assessment of interaction between the factorial interventions.

Multicenter trials should use a common rhythm-monitoring protocol, a uniform definition of the blanking period, and prespecified definitions of AF/AFL/atrial tachycardia recurrence and AF burden [3,4]. Use the same monitoring technology and minimum wear or transmission requirements across centers where possible, with central adjudication of rhythm endpoints. Biomarker sampling should occur at prespecified time points with harmonized collection conditions, assay methods, and units, and, preferably, central or quality-controlled laboratory analysis for potassium, creatinine/eGFR, renin, aldosterone, and mechanistic biomarkers. Echocardiographic LA measures and, when included, cardiac magnetic resonance or electroanatomic mapping should use core-laboratory or standardized acquisition and analysis protocols.

Renal and electrolyte safety is an ethical prerequisite, not a secondary consideration. Potassium manipulation should aim to avoid clinically important hypokalemia and hyperkalemia, not expose participants to an unvalidated high-normal target. MRA enrollment should respect established contraindications and renal/potassium thresholds, with prespecified dose-adjustment and discontinuation algorithms, rapid review of critical laboratory values, and independent safety oversight. Patients with elevated renal risk may be scientifically important but should be enrolled only with enhanced monitoring and equipoise; clinically necessary treatment of hyperkalemia, AKI, congestion, or other complications must take precedence over protocol adherence. These safeguards matter because the rhythm-prevention benefit of either intervention remains unproven.

If efficacy and safety are subsequently demonstrated, pragmatic implementation studies should test whether phenotype-guided monitoring can be delivered without excessive laboratory burden, cost, or treatment disruption. Electronic health record prompts and integration with HF, nephrology, and hypertension services may help selected high-risk patients, but should be evaluated rather than assumed to improve outcomes [1,2,6-9,11-18].

Limitations

First, direct evidence linking potassium to post-ablation recurrence is limited to a single observational association; the reported U-shaped relationship does not establish causality, identify an optimal treatment range, or demonstrate benefit from supplementation [10]. Second, direct MRA studies are few, small, heterogeneous, and mostly non-randomized, with differences in AF phenotype, renal and cardiovascular risk, ablation strategy, recurrence ascertainment, and follow-up [19,22-24]. Third, MRA treatment is strongly affected by treatment-selection bias and residual confounding because HF, CKD, resistant hypertension, potassium status, and other factors influence both prescribing and recurrence risk [6,13,17,18,29,30]. Fourth, recurrence detection varies across monitoring strategies, and binary first-recurrence endpoints may miss clinically relevant differences in AF burden [3,4].

A further limitation is that the proposed ionic-renal-aldosterone-fibrosis framework relies heavily on indirect clinical and mechanistic evidence, as direct post-ablation data are sparse [7-24]. These sources can establish biological plausibility and generate testable hypotheses, but they cannot validate causal pathways or demonstrate that modifying a particular component reduces post-ablation recurrence. Experimental findings may not translate quantitatively to the human post-ablation atrium, and AF-prevention effects observed in non-ablation populations may not apply when pulmonary vein reconnection or lesion-related mechanisms dominate. The certainty of the conceptual framework is therefore lower than its biological coherence might suggest, and its clinical utility requires prospective validation.

Citation bias is also possible. With only a small number of direct clinical studies, the narrative interpretation can be disproportionately influenced by a few positive or readily identifiable reports, while negative, unpublished, non-English, conference-only, or non-PubMed-indexed studies may be underrepresented. Hand-searching and explicit separation of direct, indirect, and mechanistic evidence reduce but do not eliminate this risk. The reliance on PubMed/MEDLINE as the primary bibliographic database further increases the possibility of database and language selection bias [25,26]. Future updates should include additional databases, trial registries, and, where feasible, non-English literature.

Finally, this is a narrative review supported by a systematic search rather than a quantitative synthesis. Heterogeneity precluded a reliable pooled estimate, and evidence may have emerged after the search date. Accordingly, the proposed potassium time-in-zone metric, phenotype-guided MRA strategy, and integrated mechanistic framework should be regarded as research hypotheses rather than validated clinical algorithms.

## Conclusions

Current evidence suggests an association between serum potassium and atrial arrhythmia recurrence after AF ablation, but the available potassium data are observational and do not establish a causal relationship, an optimal therapeutic range, or benefit from potassium supplementation. Direct evidence for MRA therapy after ablation is limited, heterogeneous, and insufficient to establish a class effect or a causal reduction in recurrence. Indirect AF-prevention and mechanistic studies support biological plausibility but cannot establish post-ablation efficacy.

Current practice should therefore remain within established guideline-supported care: identify and correct clinically important electrolyte disturbances and reversible causes, monitor renal function when indicated, and prescribe MRAs for established indications with appropriate potassium and renal surveillance. Routine potassium supplementation to a fixed high-normal target and MRA initiation solely to prevent post-ablation recurrence are not supported by current evidence. Phenotype-guided MRA therapy based on potassium instability, mineralocorticoid activity, cardiorenal phenotype, or atrial remodeling remains investigational. The proposed ionic-renal-aldosterone-fibrosis framework and potassium time-in-zone concept should be tested in adequately powered prospective studies and randomized trials using standardized rhythm monitoring, longitudinal electrolyte assessment, and rigorous renal-safety protocols before they are incorporated into post-ablation treatment recommendations.
